# Covariance modelling for randomised controlled trials

**DOI:** 10.1186/1745-6215-14-S1-O115

**Published:** 2013-11-29

**Authors:** Gilbert MacKenzie

**Affiliations:** 1 University of Limerick, Limerick, Munster, Ireland

## 

The setting of interest is that of longitudinal randomized controlled clinical trials. The question of interest is: why is it that trialists think that intervention affects the mean but not the covariance structure? Trial design and execution should be a multi-criteria activity (based on joint mean-covariance modelling) and not merely focussed on the mean (location) which, curiously, is how most trials are designed today.

Often, the covariance structure is thought to be a ‘nuisance parameter’ or at least not to be of primary ‘scientific interest’ and little effort is expended on elucidating its structure. The idea that intervention might affect the covariance structure rather than, or as well as, the mean rarely intrudes. We shall argue that these ideas are rather pass´e and that from an inferential standpoint the problem is symmetrical in both parameters. Throughout, we will distinguish carefully between joint estimation which is now relatively routine and joint model selection which is not.

At first sight the task of estimating the structure of the covariance matrix, from the data, rather than from a prespecified menu, may seem daunting, whence the idea of searching the entire covariance model space may seem prohibitive. Thus, the final demand that we conduct a simultaneous search of the Cartesian product of the mean-covariance model space may seem impossible. However, below, we shall accomplish all three tasks elegantly for a particular, but very general, class of covariance structures defined below.

We expect that the new models described will improve current practice in the trials arena.

